# Predicting the Origin of Ventricular Arrhythmia Using Acoustic Cardiography

**DOI:** 10.1038/s41598-017-15573-5

**Published:** 2017-11-14

**Authors:** Chin-Yu Lin, Shih-Lin Chang, Yenn-Jiang Lin, Li-Wei Lo, Yu-Feng Hu, Tze-Fan Chao, Fa-Po Chung, Ta-Chuan Tuan, Jo-Nan Liao, Yao-Ting Chang, Abigail Louise D. Te, Shinya Yamada, Hao-Min Cheng, Shih-Hsien Sung, Ling Kuo, Hsing-Yuan Li, Ting-Yung Chang, Hoang Quang Minh, Simon Salim, Ting-Chung Huang, Shih-Ann Chen

**Affiliations:** 1 Division of Cardiology, Department of Medicine, Taipei Veterans General Hospital, Taipei, Taiwan; 2 Institude of Clinical Medicine, Department of Medicine, National Yang-Ming University School of Medicine, Taipei, Taiwan; 3 Department of Medicine, Taipei Veterans General Hospital, Yuanshan Branch, Yilan, Taiwan

## Abstract

This study aimed to examine the relationship between measurements related to heart sounds and the origin of ventricular arrhythmia. We retrospectively evaluated 45 patients undergoing catheter ablation with contemporaneous digital acoustic cardiography of the first heart sound (S1) and the second heart sound (S2). The patients with baseline wide QRS morphology (>120 ms or aberrant conduction), heart failure, valvular heart disease, chronic pulmonary disease, and obesity were excluded. Ventricular arrhythmias from the left ventricle had an increased S1 complexity score and S1 duration in comparison to adjacent sinus beats. On the other hand, ventricular arrhythmia from right ventricle had decreased S1 complexity score and S1 duration in comparison to adjacent sinus beats. The difference of S1 (ΔS1) parameters between premature ventricular complex and sinus beat was significantly smaller in right ventricular arrhythmia group compared with and left ventricular arrhythmia group. For predicting the origin of ventricular arrhythmia, the ΔS1 duration provide better predictive accuracy (sensitivity: 100%, specificity: 100%, cutoff value: −1.28 ms) in comparison to ΔS1 complexity score (sensitivity 71.4%, specificity 75.0%, cutoff value: −0.13). The change of S1 complexity and duration determined from acoustic cardiography could accurately predict the ventricular arrhythmia origin.

## Introduction

Premature ventricular contraction (PVC) is the most common cardiac arrhythmia in patients with or without any kind of diagnosed cardiac diseases^1^. Catheter ablation is an effective therapy in the treatment of frequent PVC or ventricular tachycardia (VT). Before the advancement of cardiac mapping techniques, the origin of ventricular arrhythmia (VA) was a matter of interest but lacked relevance. When therapeutic options became available, mapping tools to locate areas responsible for arrhythmias became important. By using the mapping tool, the electrophysiologist could correlate the surface electrocardiogram (ECG) of the VA with the area in the heart at which ablation could eradicate it.

Previous study demonstrated that the reverse splitting second heart sound (S2), which required additional carotid pressure tracing tool, was associated with the origin of ectopies^2^. Transesophageal echocardiography Tissue Doppler also provided an alternative method to identify the earliest onset of myocardial excitation in the animal study^3^. However, the application of these invasive tools was not clinically practical but only reserved for study with special facility. There is no other well-validated tool to identify the origin of ventricular arrhythmia currently. The ECG still remains the simplest and cost effective non-invasive diagnostic method for determining origin of VA^4^. It is useful to localize the site of origin of VA before intervention to facilitate catheter ablation^5^. Although many ECG criteria using the vectors from 12 lead recordingscan predict VA origin, it is limited by the need to record a 12-lead surface ECG with recording of PVC. Holter monitoring with fewer leads or a single lead strip is unable to predict VA origin due to the limited information from fewer vectors.

Burggraf^6^ studied 26 left bundle branch block (LBBB) subjects and 20 normal subjects with phonocardiogram using Cambridge microphones and a Cambridge fiberoptic multichannel physiologic recorder. With LBBB the first heart sound (S1) tended to be of reduced intensity and separate mitral and tricuspid components could not be identified. Brooks *et al*.^7^ studied 20 right bundle branch block (RBBB) subjects and 67 normal subjects with simultaneous echocardiogram of valves, electrocardiogram, and phonocardiogram. Wide splitting of the S1 was observed in RBBB subjects^7,8^. These studies demonstrated the wavefront of ventricular myocardial activation affect the timing of valvular closure, which could be detected and quantified by well-designed acoustic cardiography device.

Acoustic cardiography is a cost-effective technology, which incorporates hemodynamic information and electrocardiac signals for clinical assessment^9^. Acoustic cardiography permits simultaneous acquisition of ECG and heart sound information and provides a computerized interpretation of these findings. Although, the application of heart sounds in prediction of the origin of VA has been intensively investigated, it is not clinically available because of the lack of objectivity^2,10,11^. The present study was designed to test whether acoustic cardiography could provide information in differentiating the origin of VA in clinical practice.

## Methodology

### Study participants

This was a retrospective, single center cohort study. From January 2015 to June 2017, 45 patients (31 men [68.9%], mean age 59 ± 17 years old) undergoing catheter ablation of monoform VA with contemporaneous digital acoustic cardiography were enrolled. All patients routinely underwent transthoracic two-dimensional echocardiogram and 12 lead ECG. Exclusion criteria included the echocardiographic determination of heart failure (HF) and valvular heart disease due to possible acoustic abnormalities. Patients with chronic lung diseases and obesity were also excluded, as abnormalities of pulmonary function may confound the transmission of acoustic signals. Patients were also excluded if complete RBBB or LBBB was observed (QRS duration >120 ms)^12^.

Patients were classified into two groups (Group 1, ventricular ectopic beats from right ventricle [RV]; Group 2, ventricular ectopic beats from left ventricle [LV]) based on electrophysiological study and three-dimensional electroanatomic mapping. VAs from outflow tract (OT) was documented in 15 (62.5%) patients within Group 1 and 11 (52.4%) patients within Group 2.

Institutional Review Board at Taipei Veterans General Hospital, Taipei, Taiwan approved this study without requiring patients’ informed consent (VGH-IRB Number: 2014-09-012A). All patients signed their written informed consent according to the institutional guidelines of the Taipei Veterans General Hospital. All methods were performed in accordance with the relevant guidelines and regulations.

### Acoustic cardiography

Each subject underwent the acoustic cardiographic examination before the electrophysoplogical study (AUDICOR, Inovise Medical, Inc., Portland, OR, USA). Acoustic cardiographic raw data were transferred to Inovise Medical and were analyzed by the computerized algorithm for the measurement of heart sound parameters and systolic time intervals. This algorithm has been validated by blinded interpretation of heart sound tracings by experts, and the relationship of these variables to hemodynamic measurements obtained by invasive and non-invasive methods has been previously reported^13–15^.

**Figure 1:**
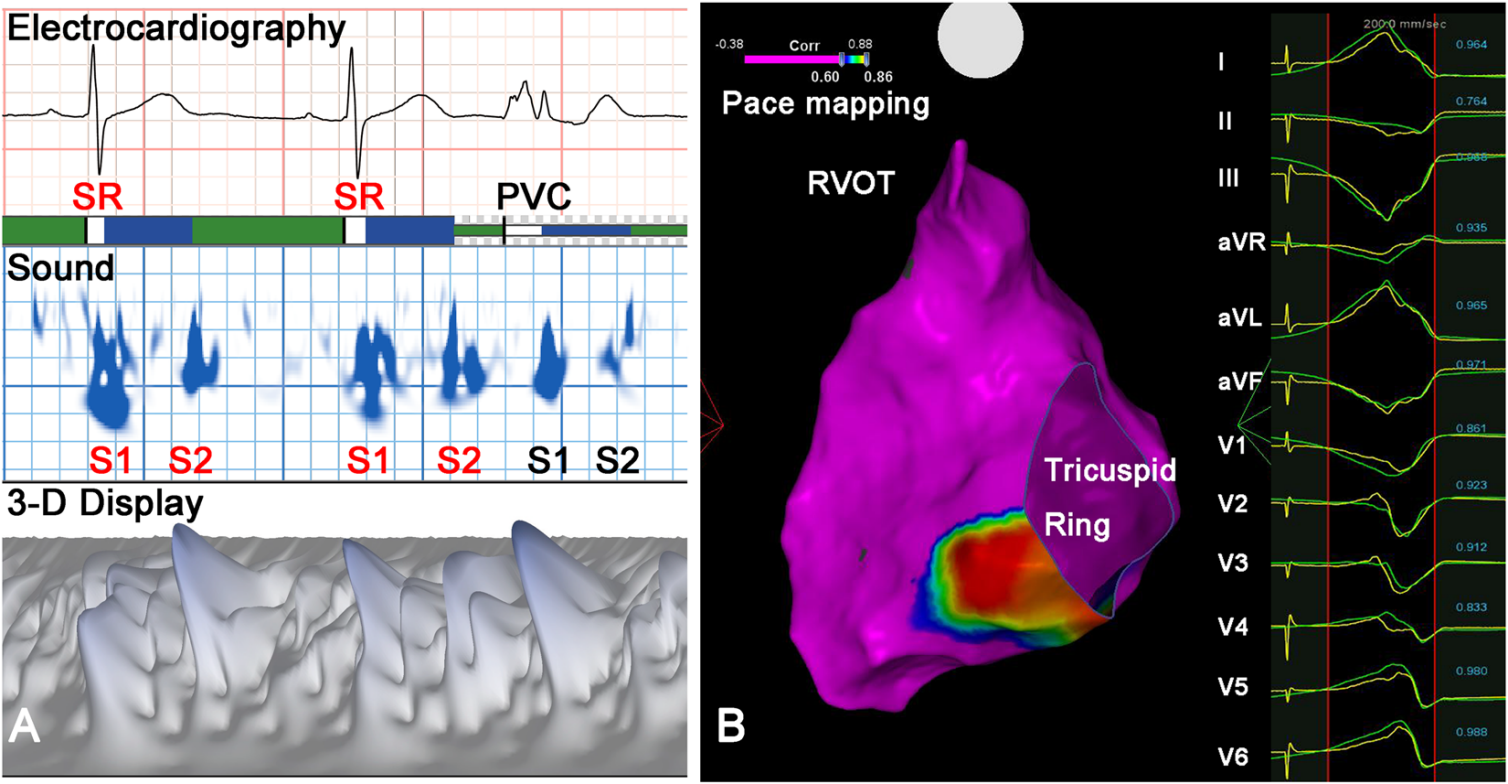
Example of acoustic cardiographic output and electroanatomic mapping in a Group 1 patient with non-outflow tract RV VA. (**A**) Acoustic cardiographic output including ECG, 2- and 3-D display of time-frequency information. The PVC S1 splitting duration and complexity were smaller than SR beats. (**B**) 3-D electroanatomic mapping with pacemapping shows a high similarity of QRS morphology located at the right ventricular basal area (red color) where catheter ablation successfully terminated the VA. 3-D, three-dimensional; ECG, electrocardiography; PVC, premature ventricular complex; SR, sinus rhythm; VA, ventricular arrhythmia.

**Figure 2:**
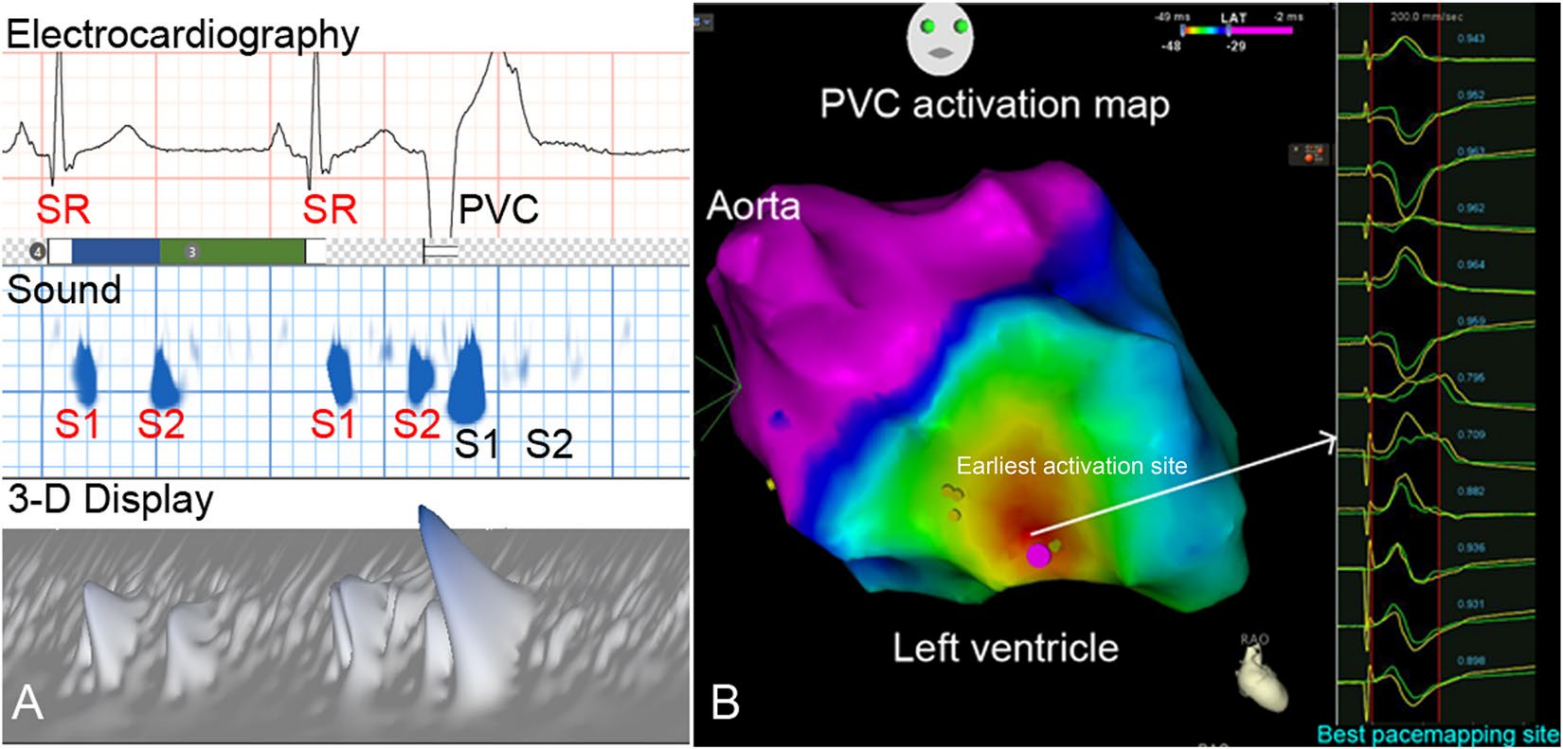
Example of acoustic cardiographic output and electroanatomic mapping of a Group 2 patient with non-outflow tract LV VA. (**A**) Acoustic cardiographic output including ECG, 2- and 3-D display of time-frequency information. The PVC S1 splitting duration and complexity were smaller than SR beats. (**B**) 3-D electroanatomic mapping with activation map shows that the earliest activation site of VA was located at the left ventricular septum area where pacemapping showed a good correlation and catheter ablation successfully terminated the VA. 3-D, three-dimensional; ECG, electrocardiography; LV, left ventricular; PVC, premature ventricular complex; SR, sinus rhythm; VA, ventricular arrhythmia.

**Figure 3:**
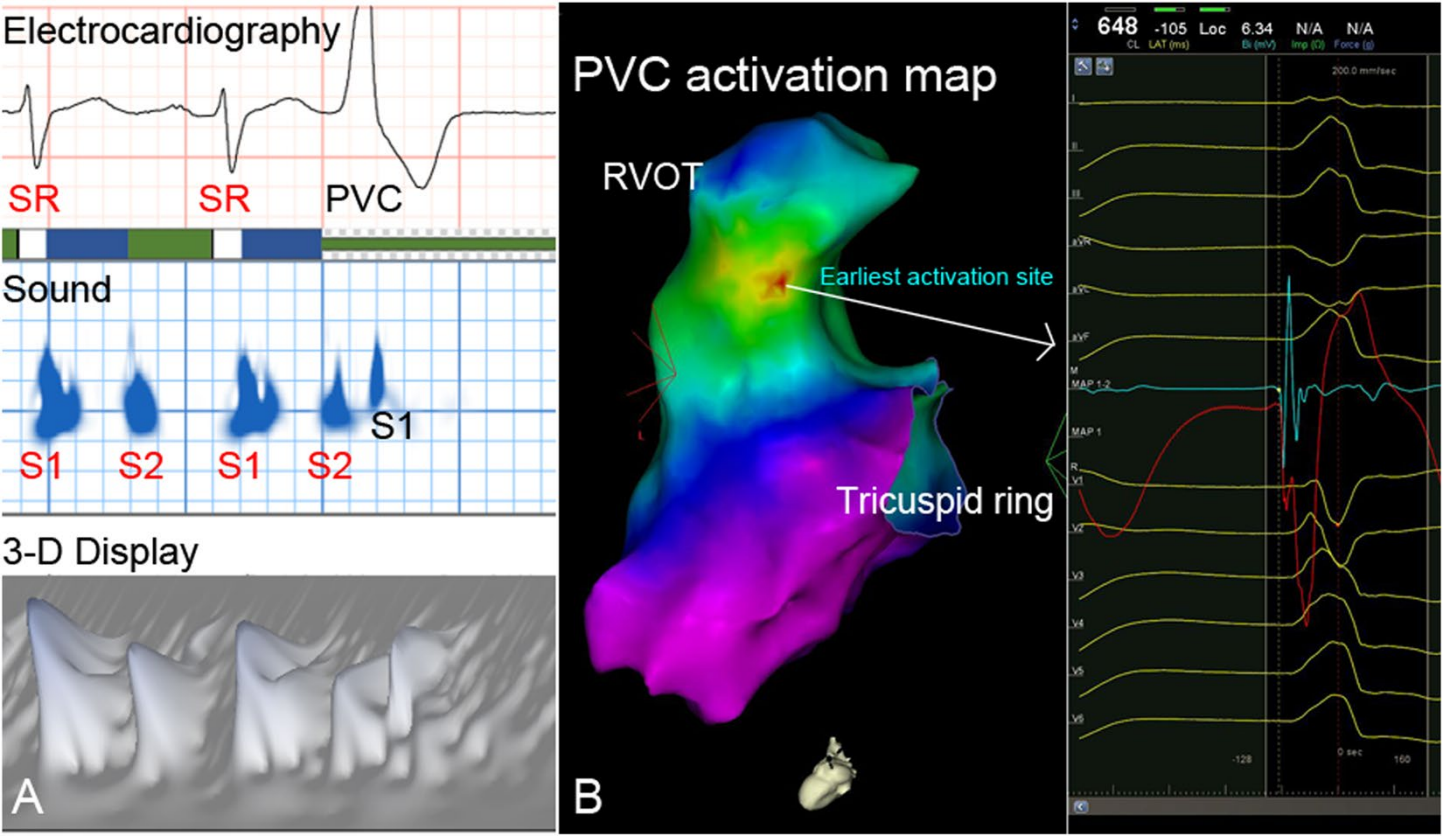
Example of acoustic cardiographic output and electroanatomic mapping of a Group 1 patient with RVOT VA. (**A**) Acoustic cardiographic output including ECG, 2- and 3-D display of time-frequency information. The PVC S1 splitting duration and complexity were smaller than SR beats. (**B**) 3-D electroanatomic mapping with activation map shows that the earliest activation site of the VA was located at the RVOT septum area where catheter ablation successfully terminated the VA. 3-D, three-dimensional; ECG, electrocardiography; PVC, premature ventricular complex; SR, sinus rhythm; RVOT, right ventricular outflow tract; VA, ventricular arrhythmia. 3-D, three-dimensional; PVC, premature ventricular complex; SR, sinus rhythm; RVOT, right ventricular outflow tract; VA, ventricular arrhythmia.

Three acoustic cardiographic characteristics of S1 and S2 were analyzed: intensity, complexity, and splitting duration. A value for heart sound intensity is generated based on the peak-to-peak amplitude of the sound and expressed in mV units. Heart sound complexity is a correlate of the auditory perception of valve sound crispness and is determined using time-frequency measures of width, intensity and frequency content of the signal, which is influenced by valve splitting or closure abnormalities. Based on spectral analysis, complexity is expressed as a dimensionless index. Splitting duration was measured by the algorithm from the onset to the end of the heart sound. (Figs 1–3A) Electromechanical activation time (EMAT) represents the time from the Q wave onset to the mitral component of the S1. EMAT reflects the time required for LV to generate sufficient force to close the mitral valve. The average acoustic cardiographic parameters were retrieved from the sinus rhythm (SR) beats and PVC beats separately. Wide QRS beats with different QRS morphology (compared to the dominant PVC) was excluded for analysis manually.

### Electrophysiological study, mapping, and radiofrequency catheter ablation

All patients previously failed treatment with at least one antiarrhythmic drug. Anti-arrhythmic drugs were discontinued for a minimum of five half-lives before radio-frequency catheter ablation (except for amiodarone). After obtaining informed consent from the patients, we performed standardized electrophysiological study in the fasting non-sedated state for the endocardial procedure and the sedated condition for epicardial approaches, respectively. In the absence of spontaneous VA, rapid ventricular pacing and/or programmed stimulation of up to three extra-stimuli were performed from the RV apex and/or the RV outflow tract (RVOT). If the VAs were not inducible, intravenous isoprenaline at 1–5 μg/min was infused in order to achieve at least a 20% increment in the heart rate. If spontaneous VAs were not inducible during pharmacological provocation, the induction protocol was repeated. The QRS morphologies of spontaneous and/or induced VAs were compared with the clinically documented VAs.

The localization of the successful ablation focus was performed using a 3D mapping system (CARTO 3 V3.2.2 Software with the User Defined Map [UDM] feature, Biosense Webster [Israel], Diamond Bar, CA, USA). During the ablation procedure inside the LV, intravenous heparin was administered in order to maintain an activated clotting time of >250 s. Pace mapping was performed in all cases using the distal bipolar electrodes at a pacing cycle length of 500 ms and stimulus amplitude of 1 mA greater than the late diastolic threshold up to a maximum output of 25 mA and pulse width of 4.0 ms. Activation mapping was intentionally performed in all cases in order to identify the earliest activation site. (Figs 1–3B).

Radiofrequency energy was delivered via an irrigated tip catheter (Navistar Thermocool, Biosense Webster, Inc., Diamond Bar, CA, USA) with a maximal power of 30–40 watts, while targeting an impedance drop of 10 Ohms, maintained for a minimum of 120 s at the site of termination or elimination in VA. Once the VA was suppressed within 30 s, additional energy was applied up to a maximum of 5 burns, followed by substrate modification of the nearby abnormal potentials, including isolated or fractionated late potentials, if present. Acute procedural success was defined as complete elimination of spontaneous or inducible VAs under the infusion of isoprenaline (up to 5 μg/min), following the same induction protocol for 30 min in order to exclude acute recurrences.

### Follow-up

Patients were followed up in the cardiology outpatient clinic with 12-lead ECGs, 24-hour Holter, monitoring, and echocardiography after the catheter ablation every 3 months for the first year, and then 6 months thereafter. For patients who could not come for outpatient follow up in our institution, they were contacted over the telephone for recurrent symptoms and recurrent arrhythmias. They were advised to visit our affiliated institutions to complete the follow-up screening, and transfer the data to the main hospital. The medical reports were obtained from those affiliated institutions. Recurrence was defined by recurrences of sustained VTs, nonsustained VTs, or greater than 1000 ventricular PVCs, as confirmed by the morphology criteria using the 24-hour Holter ECG.

### Statistical analysis

All analyses were performed using SPSS statistical software, version 20.0 (IBM Corporation, USA). Continuous data are expressed as mean standard deviation and as percentages for categorical variables. All continuous data were tested using the 1-sample Kolmogorov-Smirnov test against a normal distribution. Continuous variables were compared using the independent Student t test. For comparison of dichotomous variables, the chi-square test was used. A p value of < 0.05 was considered statistically significant. Receiver operator characteristic curves (ROC) and areas under the receiver operator characteristic curves were analyzed for the significant parameters to determine the optimal cut-off point for VA origin (Youden index).

## Results

### Study participants

The baseline characteristics of these patients are presented in the Table 1. Both groups were similar in baseline characteristics, including age, gender, medications, and clinical presentation.

**Table 1:** Baseline characteristics.

	Group 1	Group 2	P value
Number	24	21	
Age, year	59 ± 19	61 ± 15	0.686
Gender, male	16 (66.7)	15 (71.4)	0.759
Underlying disease			
Coronary artery disease	5 (20.8)	3 (14.3)	0.705
ICD implantation	4 (16.7)	3 (14.3)	0.999
Thyroid disease	0 (0.0)	3 (14.3)	0.094
Hypertension	9 (37.5)	9 (42.9)	0.767
Diabetes mellitus	3 (12.5)	4 (19.0)	0.689
Heart failure	3 (12.5)	6 (28.6)	0.267
Dyslipidemia	7 (29.2)	6 (28.6)	0.999
Symptom			
Dyspnea	4 (16.7)	2 (9.5)	0.999
Palpitation	24 (100.0)	19 (90.5)	0.212
VA characteristics			
Clinical VT	3 (12.5)	5 (23.8)	0.443
Clinical PVC	21 (87.5)	16 (76.2.5)	0.443
OT origin	15 (62.5)	11 (52.4)	0.555
Medication			
Anti-platelet	8 (33.3)	9 (42.9)	0.502
Beta-blocker	11 (45.8)	11 (52.4)	0.768
Calcium channel blocker	2 (8.3)	6 (28.6)	0.121
Propafenone	4 (16.7)	0 (0.0)	0.111
Flecainide	5 (20.8)	2 (9.5)	0.705
Amiodarone	4 (16.7)	6 (28.6)	0.476
Mexitil	6 (25.0)	1 (4.8)	0.101
ACEI or ARB	5 (20.8)	4 (19.0)	0.884
Nitrite	3 (12.5)	0 (0.0)	0.236
Aldactone	0 (0.0)	2 (9.5)	0.212

ACEI, angiotensin converting enzyme inhibitors; ARB, angiotensin receptor blockers; OT, outflow tract; PVC, premature ventricular complex; ICD, implantable cardioverter defibrillator; VA, ventricular arrhythmia; VT, ventricular tachycardia.

### Acoustic cardiography measurements of Group 1 and Group 2

There was no significant difference in acoustic cardiographic measurement of sinus beats between Group 1 and Group 2 (Table 2). Group 2 patients with VA from LV exhibited greater S1 splitting duration in comparison to Group 1 patients with VA from RV (S1 duration: 156.74 ± 28.56 vs 204.38 ± 32.49 ms, p < 0.001). The subtracted acoustic cardiographic parameters were also obtained for further analysis (PVC-SR). Group 1 patients exhibited a negative change in S1 (ΔS1) complexity and S1 duration during ventricular beats compared to SR beats. On the other hand, Group 2 patients exhibited a positive change in S1 complexity and S1 duration during ventricular beats compared to SR beats. (−0.49 ± 1.20 vs. 0.69 ± 1.24, p = 0.003 [ΔS1 complexity percentage: −0.03 ± 0.36 vs. 0.15 ± 0.41, p = 0.127]; ΔS1 duration: −18.66 ± 11.78 vs. 15.27 ± 6.80ms, p < 0.001 [ΔS1 duration percentage: −0.09 ± 0.05 vs. 0.08 ± 0.42, p < 0.001]).

**Table 2:** Acoustic cardiography measurements.

	Group 1	Group 2	P value
Number	24	21	
SR beat			
S1 intensity	10.70 ± 6.66	8.90 ± 5.56	0.333
S1 complexity	3.67 ± 1.18	3.42 ± 0.61	0.390
S1 duration	182.82 ± 22.94	189.22 ± 29.93	0.422
S2 intensity	5.19 ± 2.86	4.55 ± 1.91	0.393
S2 complexity	1.96 ± 0.69	2.44 ± 1.11	0.086
S2 duration	107.80 ± 13.63	107.74 ± 20.88	0.992
EMAT	94.91 ± 14.50	101.65 ± 14.74	0.130
PVC beat			
S1 intensity	8.51 ± 5.80	9.21 ± 5.28	0.689
S1 complexity	3.39 ± 1.02	3.79 ± 1.23	0.216
S1 duration	156.74 ± 28.56	204.38 ± 32.49	<0.001
S2 intensity	5.74 ± 5.50	5.42 ± 3.83	0.834
S2 complexity	2.07 ± 1.97	1.96 ± 0.77	0.827
S2 duration	85.84 ± 21.14	89.83 ± 34.99	0.646
EMAT	132.94 ± 21.22	136.97 ± 28.86	0.611
PVC – SR beat and [PVC – SR beat]/SR beat (% of change)			
S1 intensity	−2.10 ± 5.25	−0.09 ± 4.17	0.173
*% of change*	−0.04 ± 0.55	0.12 ± 0.50	0.323
S1 complexity	−0.49 ± 1.20	0.69 ± 1.24	0.003
*% of change*	−0.03 ± 0.36	0.15 ± 0.41	0.127
S1 duration	−18.66 ± 11.78	15.27 ± 6.80	<0.001
*% of change*	−0.14 ± 0.13	0.08 ± 0.36	<0.001
S2 intensity	−0.50 ± 1.24	0.69 ± 1.24	0.923
*% of change*	−0.19 ± 1.13	0.27 ± 1.13	0.835
S2 complexity	−0.18 ± 1.83	−0.58 ± 1.69	0.190
*% of change*	−0.03 ± 0.78	−0.09 ± 0.44	0.585
S2 duration	−20.38 ± 20.03	−19.46 ± 32.40	0.912
*% of change*	−0.20 ± 0.19	−0.17 ± 0.26	0.694

EMAT, electromechanical activation time; PVC, premature ventricular complex; S1, first heart sound; S2, second heart sound; SR, sinus rhythm.

### Acoustic cardiography measurements of OT VAs or non-OT VAs

The details of acoustic parameters between OT VAs and non-OT VAs are shown in Table 3. In the patients with LV origin VAs, there was no significant difference in acoustic cardiographic measurement of SR, PVC between OT-VAs and non-OT VAs.

**Table 3:** Acoustic cardiography measurements (OT vs. non-OT).

	LV body	LVOT	P value	RV body	RVOT	P value
Number	10	11		9	15	
***SR beat***						
S1 intensity	7.91 ± 3.90	9.79 ± 6.76	0.451	14.16 ± 9.60	8.63 ± 3.52	0.056
S1 complexity	3.64 ± 0.36	3.21 ± 0.72	0.108	3.39 ± 1.71	3.83 ± 0.74	0.398
S1 duration	183.90 ± 23.31	194.06 ± 32.33	0.451	172.68 ± 25.95	188.11 ± 18.11	0.102
S2 intensity	4.12 ± 1.19	4.95 ± 2.39	0.332	4.99 ± 4.12	5.31 ± 1.92	0.801
S2 complexity	2.09 ± 0.69	2.74 ± 1.38	0.181	1.64 ± 0.78	2.15 ± 0.57	0.115
S2 duration	111.93 ± 24.51	103.93 ± 17.25	0.394	101.35 ± 14.57	110.46 ± 9.57	0.112
EMAT	96.55 ± 10.74	106.01 ± 14.34	1.05	99.07 ± 21.25	92.42 ± 8.36	0.287
***PVC beat***						
S1 intensity	9.64 ± 5.84	8.93 ± 4.65	0.576	8.61 ± 8.67	8.84 ± 2.95	0.946
S1 complexity	3.97 ± 1.45	3.63 ± 1.05	0.549	2.91 ± 1.21	3.60 ± 0.74	0.097
S1 duration	199.76 ± 24.92	208.58 ± 38.88	0.548	143.03 ± 22.82	161.57 ± 26.35	0.125
S2 intensity	4.94 ± 2.04	5.86 ± 5.02	0.615	3.93 ± 2.59	6.69 ± 6.42	0.112
S2 complexity	1.87 ± 0.74	2.06 ± 0.84	0.620	1.10 ± 1.57	2.65 ± 2.26	0.110
S2 duration	101.16 ± 40.60	80.03 ± 23.16	0.168	77.05 ± 15.94	89.12 ± 19.85	0.236
EMAT	125.59 ± 21.49	147.21 ± 37.77	0.104	133.03 ± 26.12	132.88 ± 18.25	0.988
***PVC – SR***						
S1 intensity	1.20 ± 2.98	−1.17 ± 4.09	0.012	−5.55 ± 5.19	0.29 ± 3.90	0.007
*% of change*	0.05 ± 0.48	−0.50 ± 0.44	0.104	−2.14 ± 0.34	−0.54 ± 0.60	0.504
S1 complexity	0.35 ± 1.51	1.00 ± 0.90	0.240	−0.48 ± 1.04	−0.23 ± 1.25	0.198
*% of change*	0.11 ± 0.42	0.19 ± 0.42	0.655	−0.09 ± 0.31	−0.02 ± 0.38	0.184
S1 duration	15.97 ± 5.98	14.64 ± 7.71	0.667	−29.65 ± 13.70	−26.54 ± 10.86	0.605
*% of change*	−0.09 ± 0.03	−0.08 ± 0.04	0.461	−0.18 ± 0.16	−0.11 ± 0.10	0.104
S2 intensity	0.58 ± 2.39	0.72 ± 5.30	0.945	−1.06 ± 4.01	1.38 ± 6.44	0.089
*% of change*	−0.20 ± 0.64	−0.32 ± 1.47	0.823	−0.24 ± 0.39	0.49 ± 1.38	0.104
S2 complexity	−0.27 ± 1.03	−0.92 ± 2.26	0.445	−0.54 ± 0.56	0.69 ± 2.23	0.123
*% of change*	−0.07 ± 0.37	−0.11 ± 0.53	0.854	−0.28 ± 0.47	0.22 ± 0.88	0.104
S2 duration	−9.95 ± 32.41	−28.02 ± 31.53	0.235	−24.30 ± 20.25	−18.28 ± 19.61	0.358
*% of change*	−0.09 ± 0.25	−0.25 ± 0.26	0.178	−0.24 ± 0.20	−0.18 ± 0.18	0.204

EMAT, electromechanical activation time; LV, left ventricle; LVOT, left ventricular outflow tract; OT, outflow tract; PVC, premature ventricular complex; RV, right ventricle; RVOT, right ventricular outflow tract; S1, first heart sound; S2, second heart sound; SR, sinus rhythm.

In the patients with LV origin VAs, the subtracted acoustic cardiographic parameters (PVC-SR) in non-OT VAs exhibited a positive change in S1 intensity than that in the OT group (LV: non-OT vs OT: 1.20 ± 2.98 vs. −1.17 ± 4.09, *p* value = 0.012).

In the patients with RV origin VAs, the subtracted acoustic cardiographic parameters (PVC-SR) in non-OT VAs exhibited a negative change in S1 intensity than that in the OT group (LV: non-OT vs OT: −5.55 ± 5.19 vs. 0.29 ± 3.90, *p* value = 0.007).

### Electrophysiological mapping and acoustic findings

The PVC origins were confirmed by the electrophysiological mapping and successful ablation site. The localization of the successful ablation focus was performed using a 3-dimentional mapping system. Pace mapping and/or activation mapping were performed in order to identify the site of origin of the VA.

Radiofrequency energy was delivered via an irrigated tip catheter at the origin site of VA. Procedural success was defined as complete elimination of spontaneous or inducible VAs under the infusion of isoprenaline (up to 5 μg/min), following the same induction protocol for 30 min. Figures 1–3 shows examples of VA patient who received acoustic cardiography followed by catheter ablation. Acoustic cardiographic output including ECG, 2- and 3-dimensional display of time-frequency information. In the Group 1 patients, the PVC S1 splitting duration was smaller than SR beats (Figs 1A and 3A). Three- dimensional electroanatomic mapping and pacemapping showed that the PVCs exit were located at right lower free wall and RV outflow tract respectively (Figs 1B and 3B). In the Group 2 patients, the PVC S1 splitting duration was longer than SR beats (Fig. 2A). Three-dimensional electroanatomic mapping and pacemapping showed the PVC exit was located at LV septum (Fig. 2B). PVCs were eliminated successfully by catheter ablation.

### Value of quantitative acoustic variables for the detection of VAs origins

**Figure 4:**
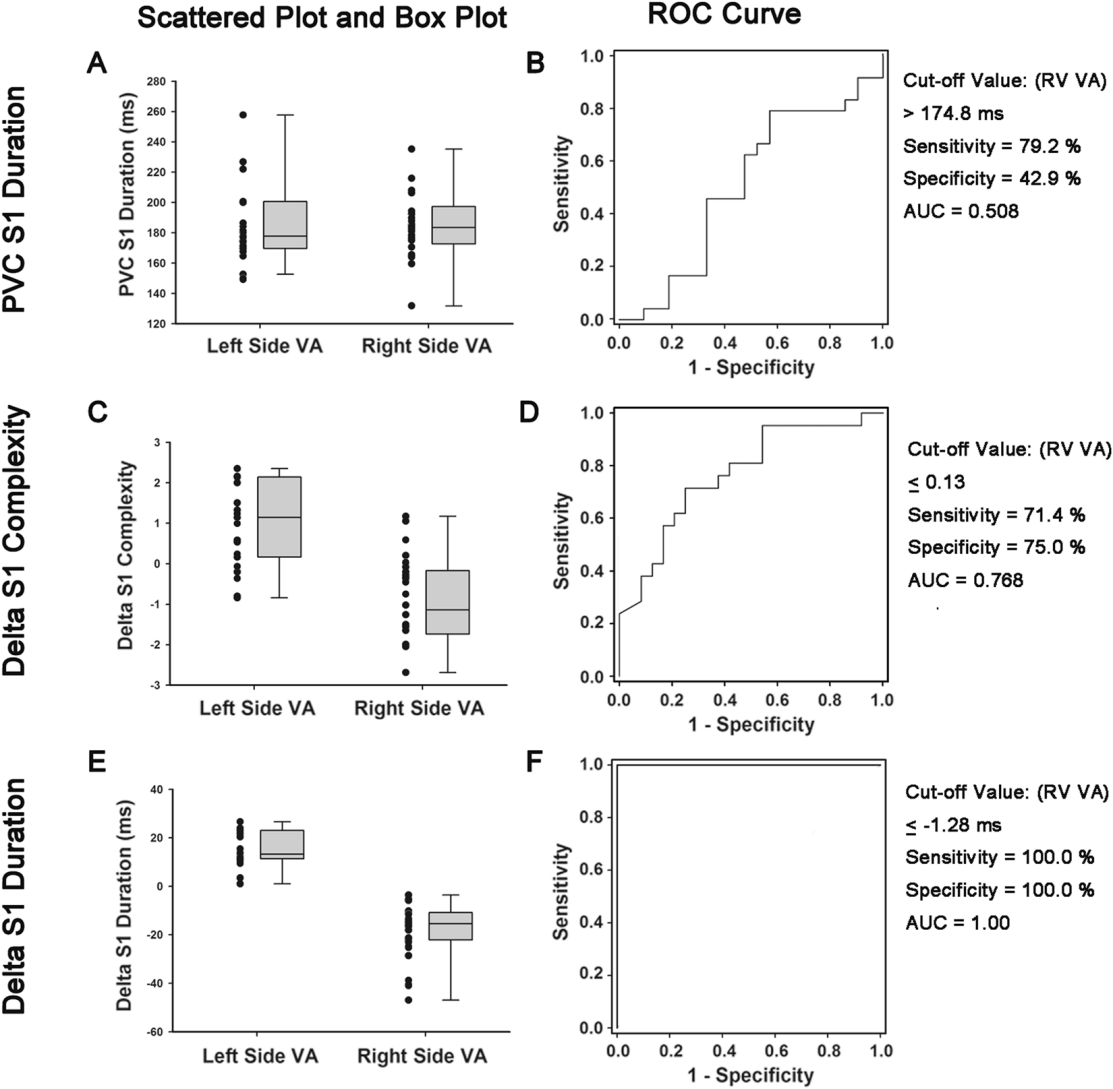
Scatter plots, Box plots, and ROC curve. (**A**) Box plot and scatter plot demonstrating the differences and the distribution of PVC S1 Duration of patients with VA originating from different chambers. (**B**) The ROC curve analysis of the PVC S1 Duration for predicting the VAs origin. (**C**) Box plot and scatter plot demonstrating the differences and the distribution of [PVC-SR] S1 Complexiity of patients with VA originating from different chambers. (**D**) The ROC curve analysis of the [PVC-SR] S1 Complexiity for predicting the VAs origin. (**E**) Box plot and scatter plot demonstrating the differences and the distribution of [PVC-SR] S1 Duration of patients with VA originating from different chambers. (**F**) The ROC curve analysis of the [PVC-SR] S1 Duration for predicting the VAs origin. AUC, area under the curve of ROC; LV, left ventricle; PVC, premature ventricular complex; ROC, receiver operating characteristic curve; RV, right ventricle; S1, first heart sound; S2, second heart sound; SR, sinus rhythm; VA, ventricular arrhythmia. Box plot explanation: upper horizontal line of box, 75th percentile; lower horizontal line of box, 25th percentile; horizontal bar within box, median; upper horizontal bar outside box, 90thpercentile; lower horizontal bar outside box, 10th percentile.

We investigated the value of PVC S1 duration, ΔS1 complexity, and ΔS1 duration for the detection of VA origin (right side or left side) by constructing ROC curves to assess the predictive performance. As shown in Fig. 4 the area under ROC curve for PVC S1 duration, ΔS1 complexity, and ΔS1 duration was 0.508, 0.768, and 1.00 respectively. The optimal cut-offs of PVC S1 duration, ΔS1 complexity, and ΔS1 duration for the discrimination of VA origin was 174.8 ms (sensitivity 79.2%, specificity 42.9%), 0.13 (sensitivity 71.4%, specificity 75.0%) and −1.28 ms (sensitivity 100.0%, specificity 100.0%) respectively.

Regarding the traditional ECG criteria used to discriminate origin of VA, 24 (100.0%) RV VAs were presented with LBBB morphology, 17 (81.0%) LV VAs were presented with RBBB morphology. Four (19.0%) LV VAs were presented with LBBB morphology with early precordial transition before V2. The overall sensitivity and specificity was inferior to ΔS1 duration in the present study.

### OT-VA vs. non-OV VA in left ventricle and right ventricle

**Figure 5:**
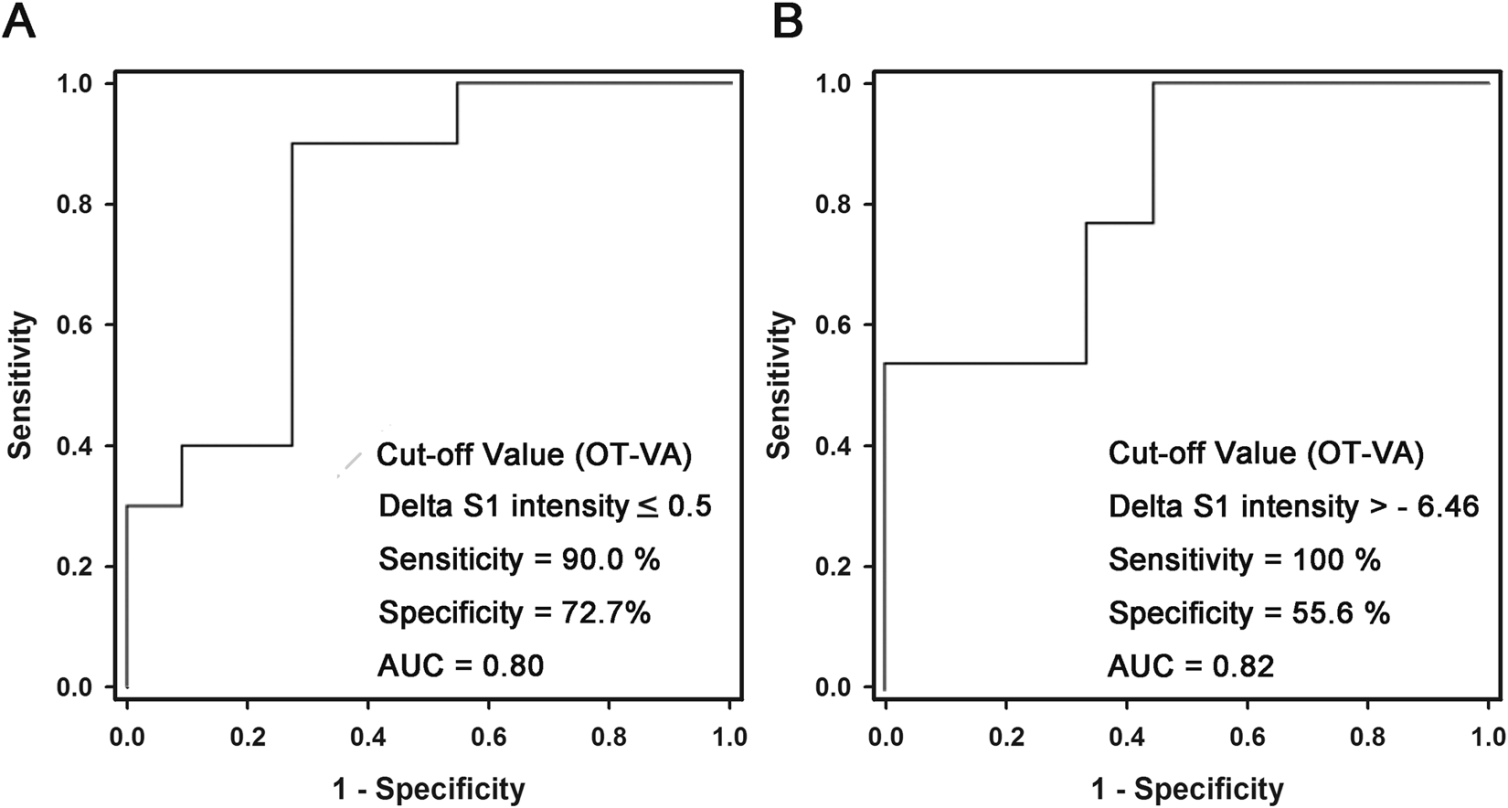
ROC curve analysis for predicting the OT VAs or non-OT Vas. (**A**) The ROC curve analysis of the [PVC-SR] S1 intensity for predicting the area of origin in the left ventricle. (**B**) The ROC curve analysis of the [PVC-SR] S1 intensity for predicting the area of origin in the right ventricle. AUC, area under the curve of ROC; PVC, premature ventricular complex; ROC, receiver operating characteristic curve; S1, first heart sound; S2, second heart sound; SR, sinus rhythm; VA, ventricular arrhythmia.

In the patients with LV VAs, we investigated the value of ΔS1 intensity for the detection of OT VAs by constructing ROC curves to assess the predictive performance. As shown in Fig. 5A, the area under ROC curve for ΔS1 intensity was 0.80. The optimal cut-off value was 0.50 with sensitivity 90.0%, specificity 72.7%.

In the patients with RV VAs, we also investigated the value of ΔS1 intensity for the detection of OT VAs by constructing ROC curves to assess the predictive performance. As shown in Fig. 5B, the area under ROC curve for ΔS1 intensity was 0.82. The optimal cut-off value for ΔS1 intensity was −6.46 (Sensitivity 100%, Specificity 55.6%).

### Clinical follow-up

After a median follow-up of 16 ± 6 months (range 3 to 32 months), VA recurrences were documented in 4 patients (8.9%) after a mean follow-up of 7.5 (4–14) months. There was no statistical significance in the VA recurrences between the two groups (1 in Group 1 and 3 in Group 2, P value = 0.234). The QRS morphology of recurrent VA was similar to that before the catheter ablation in the 4 patients and repeated catheter ablation at the similar location eliminated the recurred VA smoothly.

## Discussion

### Acoustic profiles and ventricular ectopies

To the best of our knowledge, this is the first study to evaluate the value of acoustic profile on prediction of origin of ventricular arrhythmia. With LBBB the S1 tends to be of reduced intensity as shown in a previous study^6^. The reduced intensity of the S1 in LBBB is caused mainly by the decreased amplitude of the mitral valve excursion at the onset of left ventricular contraction. In our study, LBBB morphology PVC (RV origin) demonstrated non-significant reduced intensity, decreased complexity and shortened S1 slitting duration. Our result echoed the previous findings that reduced complexity and splitting duration of S1 in PVC with RV origin is caused mainly by the decreased amplitude of the mitral valve excursion at the onset of LV contraction and the decreased tension on the closed mitral valve resulting from the slow rate of LV pressure rise at the onset of the S1^16^.

In our study population, 4 patients had left ventricular outflow tract (LVOT) VAs with LBBB morphology. This finding suggested that the acoustic parameter might provide better predictive value than traditional Holter with 3 ECG channels.

With RBBB the S1 tends to be widely spitted with a preserved amplitude and a larger late component. as shown in a previous study^7,8^. In this study, RBBB morphology PVC (LV origin) was characterized by increased S1 duration and complexity, which were compatible with previous findings.

### Acoustic profile and electrophysiology

The acoustic profiles of S1 and S2 during sinus rhythm didn’t significantly differ between Group 1 and Group 2. It is well understood that the intensity of precordial heart sounds is influenced by anatomic factors such as body surface area, characteristics of the thoracic cage and cardiac axis^17^. To eliminate the individual anatomic difference, we also evaluated the difference of acoustic profiles between PVC and SR beats. We observed that the complexity and splitting duration of S1 were significantly increased in left side PVCs compared to that in SR. Reversely, reduced complexity and splitting duration of S1 were found in right side PVCs compared to that in sinus beats. The difference of PVC and SR (PVC-SR) in S1 duration was shown to be a better parameter in differentiating VA origin.

To the best of our knowledge, there was no study to evaluate the acoustic difference between OT-VA and non-OT VAs. Our presented study demonstrates that the OT-VA and non-OT VA had different acoustic parameter as S1 intensity in LV VA and S2 duration and S2 complexity in RV VA. Further prospective studies with systemic approaches are warranted to prove this finding.

### The mechanism of ΔS1 duration

The S1 is produced by vibrations generated by the closure of the mitral and the tricuspid valves. There is normal asynchrony in the closure of mitral and tricuspid valves, the mitral closure preceding tricuspid closure by 20 to 30 msec^18^. Ventricular ectopy from LV activated LV myocardium and initiate mitral valve closure first. Late onset of RV contraction secondary to a mean 25.7 msec transeptal conduction time^19^ contributed to the prolonged S1 during LV PVC, which was caused by delayed onset of the right ventricular pressure pulse and tricuspid closure. Our present study demonstrated a positive change of S1 splitting duration of LV PVC in comparison to SR, which echoed the previous concept of hemodynamic and electrophysiological aspects.

In the previous study, the atrioventricular valve closure times during LBBB morphology beats^6^, mitral valve closure occurred earlier than tricuspid valve in 27% of the subjects. Some hemodynamic studies have been conducted to determine the delay in onset of left ventricular contraction in LBBB morphology beats and the reversed splitting was not consistently observed on the phonocardiograms^20–22^. Our present study revealed a negative change of S1 duration with large standard deviation (−18.66 ± 11.78 ms) of RV PVC in comparison to SR beats, which echoed the previous reports.

In view of electrophysiological hemodynamic points, the LV PVC exaggerated the normal asynchrony through transseptal conduction time. On the other hand, the RV PVC shortened or reversed the naturally splitting sequence of atrioventricular valve closure and produced a relatively negative change of S1 during comparing to LV PVCs.

### Clinical implication

Conventional Holter examination could only provide the information regarding the presence of arrhythmia. In addition to the ECG strip, the acoustic cardiography was automatically analyzed for the measurement of heart sound parameters, which effectively provided the information of potential VA origin. The acoustic cardiographic examination can be used to determinate the dominant PVC origin with our documented 12-lead ECG or symptomatic PVC origin and guide the clinical decision making before and during interventional electrophysiologic procedures. After successful ablation, the acoustic cardiography could provide the information regarding the pattern of recurrence (same origin or not). Before our present study, invasive electrophysiological study and 12 leads surface ECG were the only clinically available methodology in determining VA origin. The clinical value of conventional ECG was limited by the short recording time (10 seconds strip with 2.5 seconds in each lead). The acoustic cardiography not only provided the information of the presence of arrhythmia but also the origin of ventricular ectopic beats. Based on this additional information, clinician can be informed of the potential benefit and procedure related complication in detail.

## Limitations

Several limitations merit consideration. First, the sample size was relatively small, which provided a weak power for the generalization of the conclusion. We adjusted for possible inter-subject anthropomorphic differences by calculating the difference between PVC and SR beats. Second, this study is limited by its retrospective nature. Third, although it’s clinical value has been established in previous literatures, acoustic cardiography is easily affected by environmental interference. Carefully review and manual editing by experienced technician were required. Fourth, although the change of S1 complexity, and S1 splitting duration displayed potential predictive utility for the discrimination of VA origin, the study cohort was preselected to elucidate the physiological underpinnings of transmitted heart sounds, and thus patients with valvular abnormalities and conduction disorders were excluded. Fifth, the clinical value of acoustic cardiography for VA in the patients with structural heart disease or valvular heart disease was not clear because of the study design. Additionally, this present study included patients with distinctive VA origin from RV and LV. Various impact of activation wavefront might affect our observation on the heart sound change. Further prospective trial with different cohort and larger samples is warranted for validation and confirmation.

## Conclusion

The difference of S1 splitting duration between SR and PVC could accurately predict the chamber of VA origin. Further acoustic analysis might help differentiate the OT VA and non-OT VA. Increased PVC S1 duration compared to that of SR beats was altered in the left side VA group. In the group with LV VA, OT origin VA was presented with negative change in S1 intensity during PVC. In the RV VA, OT origin VA was presented with positive change in S1 intensity during PVC. These findings extend our mechanistic understanding of the relationship between ventricular arrhythmia and the precordial acoustic profile. Further prospective study is warranted for applying the acoustic data in differentiating the location of VA.
